# New Appliances and Things Medical

**Published:** 1899-06-17

**Authors:** 


### NEW APPLIANCES AND THINGS MEDICAL.

[We shall be glad to receive, at our Office, 28 & 29, Southampton Street, Strand, London, W.C., from the manufacturers, specimens of all new preparations and appliances which may be brought out from time to time.]

### MIGRÄNIATOR.

(Schönlin and Co., München, Germany.)

The Migräniator consists of two soft pads attached to the two ends of adjustable steel springs, which are so shaped as to fit comfortably to the head and bring a certain amount of pressure to bear on selected points—these usually are the two sides of the temple. Its action is, in fact, very much the same as an ordinary truss, from which it differs by the adjustability of the springs. It has been found of considerable use in cases of migraine, nervous headache, and other forms of cephalic neuralgia. There is a tendency at the present day to adopt physical as opposed to medicinal methods of treatment, especially for functional nervous disorders, and it is to meet this demand that the migräniator has been devised for use in that class of functional nervous diseases which manifest themselves in the region of the cephalic dome and upper part of the face. The instrument is comfortable and convenient to wear, without interfering with the continuance of ordinary business.

### OZONIC INHALER.

(W. Martindale, 10, New Cavendish Street, London, W.)

The ozonic inhaler consists of a flask-like glass vessel, open at both ends. One end is flattened and shaped so as to form a convenient mouthpiece, the other end admits a current of air, and the bulbous enlargement between the two openings is stuffed with wood shavings for the receipt of the medicament. For use, a few drops of the medicament, whether it be eucalyptus oil, carbolic smelling salts, or other substance, are poured through the wide opening, the mouthpiece is placed between the lips, expiration being carried in through the nose. For the treatment of nasal and pharyngeal catarrhs, hay fever, and allied conditions, this cheap and cleanly little device should be found most serviceable.

